# CDC42 is required for epicardial and pro-epicardial development by mediating FGF receptor trafficking to the plasma membrane

**DOI:** 10.1242/dev.147173

**Published:** 2017-05-01

**Authors:** Jingjing Li, Lianjie Miao, Chen Zhao, Wasay Mohiuddin Shaikh Qureshi, David Shieh, Hua Guo, Yangyang Lu, Saiyang Hu, Alice Huang, Lu Zhang, Chen-leng Cai, Leo Q. Wan, Hongbo Xin, Peter Vincent, Harold A. Singer, Yi Zheng, Ondine Cleaver, Zhen-Chuan Fan, Mingfu Wu

**Affiliations:** 1 Department of Molecular and Cellular Physiology, Albany Medical College, Albany, NY 12208, USA; 2 Institute of Translational Medicine, Nanchang University, Nanchang 330031, China; 3 School of Life Sciences, Nanchang University, Nanchang 330031, China; 4 Developmental and Regenerative Biology, Mount Sinai Hospital, New York, NY 10029, USA; 5 Department of Biomedical Engineering, Rensselaer Polytechnic Institute, 110 8th street, Biotech 2147, Troy, NY 12180, USA; 6 Division of Experimental Hematology and Cancer Biology, Cincinnati Children's Hospital Medical Center, Cincinnati, OH 45229, USA; 7 Molecular Biology, UT Southwestern, Dallas, TX 75390, USA; 8 International Collaborative Research Center for Health Biotechnology, Tianjin University of Science and Technology, Tianjin 300457, China

**Keywords:** Epicardium development, Pro-epicardial cells, FGFR1 trafficking, CDC42, FGF2 signaling, Mouse

## Abstract

The epicardium contributes to multiple cardiac lineages and is essential for cardiac development and regeneration. However, the mechanism of epicardium formation is unclear. This study aimed to establish the cellular and molecular mechanisms underlying the dissociation of pro-epicardial cells (PECs) from the pro-epicardium (PE) and their subsequent translocation to the heart to form the epicardium. We used lineage tracing, conditional deletion, mosaic analysis and ligand stimulation in mice to determine that both villous protrusions and floating cysts contribute to PEC translocation to myocardium in a CDC42-dependent manner. We resolved a controversy by demonstrating that physical contact of the PE with the myocardium constitutes a third mechanism for PEC translocation to myocardium, and observed a fourth mechanism in which PECs migrate along the surface of the inflow tract to reach the ventricles. Epicardial-specific *Cdc42* deletion disrupted epicardium formation, and *Cdc42* null PECs proliferated less, lost polarity and failed to form villous protrusions and floating cysts. FGF signaling promotes epicardium formation *in vivo*, and biochemical studies demonstrated that CDC42 is involved in the trafficking of FGF receptors to the cell membrane to regulate epicardium formation.

## Abstract (precis)

**Highlighted article:** During epicardial formation in mice, four different mechanisms of pro-epicardial cell translocation to the myocardium can be identified, with CDC42 playing a key role.

## INTRODUCTION

The epicardium, which consists of a single layer of squamous epicardial cells (ECs) that covers the heart, is the major source of coronary smooth muscle cells and cardiac fibroblasts (Acharya et al., 2012; Dettman et al., 1998; Gittenberger-de Groot et al., 1998; Lie-Venema et al., 2007; Manner, 1999; Mikawa and Fischman, 1992; Mikawa and Gourdie, 1996; Vrancken Peeters et al., 1999). In addition to contributing to cardiac lineages during development, it is also involved in cardiac regeneration under the stress of injury by secreting growth factors and differentiating to cardiac lineage cells (Lepilina et al., 2006; Russell et al., 2011; van Wijk et al., 2012; Wang et al., 2015; Zhou et al., 2011). Despite its essential role in cardiac development and regeneration, the cellular mechanisms underlying epicardium formation from the pro-epicardium (PE) are not fully understood, and the molecular signaling pathways and underlying genetic mechanisms remain unclear.

The PE is a transient bunch of grapes-like structure located at the surface of the sinus venosus near the venous pole of the embryonic heart (Kuhn and Liebherr, 1988; Manasek, 1969; Mikawa and Gourdie, 1996; Viragh and Challice, 1981). The PE consists of diverse progenitor cells that give rise to different cardiac lineages depending on the region of the PE from which the cells are derived (Katz et al., 2012; Keith and Bolli, 2015). In mouse, at approximately embryonic day (E) 9.0, pro-epicardial cells (PECs) dissociate from the PE, translocate across the pericardial cavity, and then attach to the heart surface. Upon reaching the heart, the cells spread over and eventually envelop the heart as a simple squamous epithelium known as the epicardium. A subset of ECs will then undergo epicardial-mesenchymal transition (EMT) and differentiate into different cardiac cell types during cardiac development and cardiac regeneration (Cai et al., 2008; Dettman et al., 1998; Mikawa and Fischman, 1992; Mikawa and Gourdie, 1996; Wu et al., 2010; Zhou et al., 2011, 2008).

How PECs reach the heart is not fully understood, but it is believed to be species specific. In avian development, the PE extends bleb-like villi that form a transient tissue bridge that links PECs to specific sites of the dorsal surface of the looping heart in a BMP-dependent manner (Ishii et al., 2010). In mammalian embryos, the PECs are released as free-floating cysts, which translocate across the pericardial cavity to reach the heart and form epicardial islands on the ventricular surface (Hirose et al., 2006; Sengbusch et al., 2002). These epicardial islands then spread to form isolated patches of epicardial sheets that subsequently coalesce to form the epicardium (Komiyama et al., 1987; Viragh and Challice, 1981). However, it was also demonstrated in mouse that PECs translocate to the heart by direct PE contact with the myocardium (Rodgers et al., 2008). The approaches that we applied in this study, such as PE lineage tracing and whole-heart three-dimensional (3D) imaging, were designed to reveal these mechanisms in detail and resolve the controversy.

A potential cellular mechanism regulating PEC dissociation and translocation is cellular polarity. Previous studies have shown that Par3 is required for PEC dissociation by establishing PEC polarity and interpreting the polarity cues from cell-cell and cell-extracellular matrix interactions (Hirose et al., 2006). The cell division control protein CDC42 is a small GTPase of the Rho family that is essential in establishing cell polarity (Cau and Hall, 2005). In responding to different cellular signals, the ubiquitously expressed CDC42 cycles between a GDP-bound inactive state and a GTP-bound active state through the actions of GTPase activating proteins, guanine nucleotide exchange factors, and guanine nucleotide dissociation inhibitors (Bernards, 2003; Etienne-Manneville and Hall, 2002; Jaffe and Hall, 2005; Olofsson, 1999; Schmidt and Hall, 2002). CDC42 regulates angiogenesis via VEGFR2 (KDR) shedding (Jin et al., 2013) and cytoskeletal support of endothelial cell adhesion (Barry et al., 2015), and regulates *Drosophila* and mouse heart morphogenesis (Li et al., 2017; Vogler et al., 2014). CDC42 provides an anti-hypertrophic switch in the adult heart (Maillet et al., 2009) and regulates adult heart functions synergistically with *Nkx2.5* across species (Qian et al., 2011). Whether CDC42 is involved in epicardium development has not been investigated.

In this study, we found that murine PECs reach the heart via villous projections, cyst formation, and through PE directly contacting heart as previously reported (Rodgers et al., 2008); moreover, we report a fourth mechanism in which PECs migrate along the surface of the inflow tract to reach the ventricle. When *Cdc42* was deleted via *Tbx18^Cre/+^* in the PE to generate a conditional knockout (CKO), the formation of floating cysts and villous projections was disrupted. The PECs of the CKO did migrate along the inflow tract toward the ventricle, and translocated by direct contact between the PE and the heart. However, these two mechanisms in the CKO did not result in complete formation of the epicardium, which eventually caused embryonic lethality. Further mosaic studies in the inducible CKO (iCKO) showed that deletion of *Cdc4*2 in PECs impaired cellular dynamics. We additionally found that FGF2 regulates PEC dissociation and translocation in a CDC42-dependent manner, and that CDC42 is required for FGF receptor trafficking to the cell membrane. This study indicates that CDC42 regulates multiple steps in PE development, including establishing PEC polarity and controlling the trafficking of FGFR1 to the cell membrane.

## RESULTS

### CDC42 is required for epicardium development

**Fig. 1.**
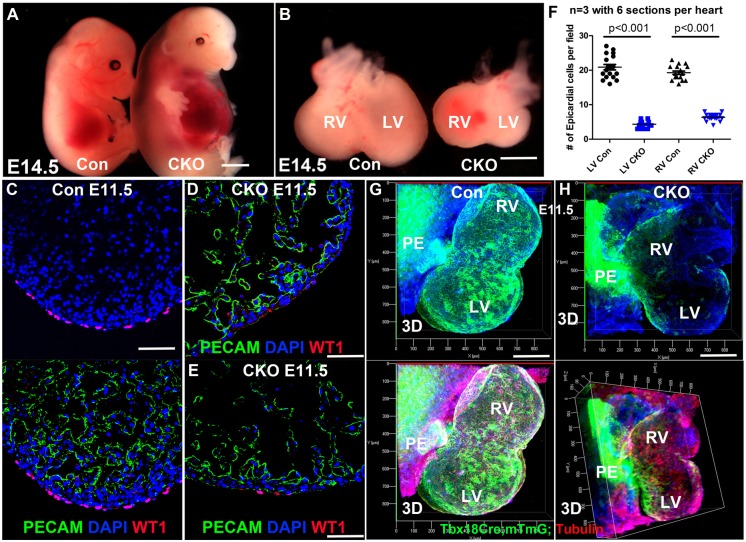
**CDC42 is required for epicardium development.** (A,B) A representative CKO mouse embryo at E14.5 displayed edema (A) and had a smaller heart with abnormal morphology compared with the control heart (B). (C-E) The epicardium of the control heart at E11.5 was fully covered with WT1^+^ cells (C), whereas the CKO heart was not, showing absence of WT1^+^ cells (D) or sparse distribution of WT1^+^ cells (E) in some regions. (F) At E12.5, the CKO has significantly fewer cells per field in both the LV and RV (F). (G,H) *Tbx18^Cre/+^; Cdc42^fl/+^; mTmG* (Con) and CKO embryos at E11.5 were whole-mount stained for α-tubulin and PECAM, then cleared, imaged and reconstructed using Imaris. The epicardium of the control heart was fully covered with GFP-labeled ECs (G), whereas only the dorsal surface of the CKO heart was partially covered (H). RV, right ventricle; LV, left ventricle; 3D, three-dimensional reconstruction. An unpaired two-tailed Student's *t*-test was used to determine statistical significance. Scale bars: 200 μm in A,B; 100 μm in C-E,G,H.

Our previous work has shown that ECs display polarity (Wu et al., 2010). To further study cell polarity in epicardium development, *Cdc42*, a gene encoding a small GTPase that is required to establish cell polarity (Etienne-Manneville, 2004), was deleted specifically in the epicardium via *Tbx18^Cre/+^*. Tbx18 is expressed in ECs, PECs and some cardiomyocytes in the septum (Cai et al., 2008; Christoffels et al., 2009). We examined the *Tbx18^Cre^; mTmG* hearts at E9.5 and E10.5 and found that GFP labeled the ECs, PECs and very few cells in the wall of the inflow tract in some hearts. Since we only quantify the labeled cells that localize to the surface of the heart, the PE, or inside the pericardial lumen, the expression of *Tbx18^Cre^* in the inflow tract and in some septal cardiomyocytes at later stages does not affect the quantification and conclusions drawn in this study. The *Tbx18^Cre/+^; Cdc42^fl/fl^* (CKO) embryos displayed edema at E14.5 (Fig. 1A), and their hearts were smaller and showed abnormal morphology compared with controls (Fig. 1B). The myocardium was significantly thinner, with the compact zone being 11±3 μm in CKO and 24±5 μm in control at E11.5 (*n*=3 hearts for each genotype, *P*<0.001). The CKO embryos started to die at ∼E14.5 (Table 1). The CKO heart was not fully covered by epicardium based on the expression of WT1 (Fig. 1C-H) and Raldh2 (Aldh1a2) (Fig. S1A), markers for epicardium (Moss et al., 1998; Zhou et al., 2008), with some regions completely lacking WT1^+^ cells (Fig. 1D) and other regions showing sparse WT1^+^ or Raldh2^+^ cell coverage at E11.5 (Fig. 1E , Fig. S1A). Similarly, the CKO heart at E12.5 displayed significantly fewer WT1^+^ cells (Fig. 1F). These results imply that the CKO displayed an epicardium development defect.

**Table 1.**
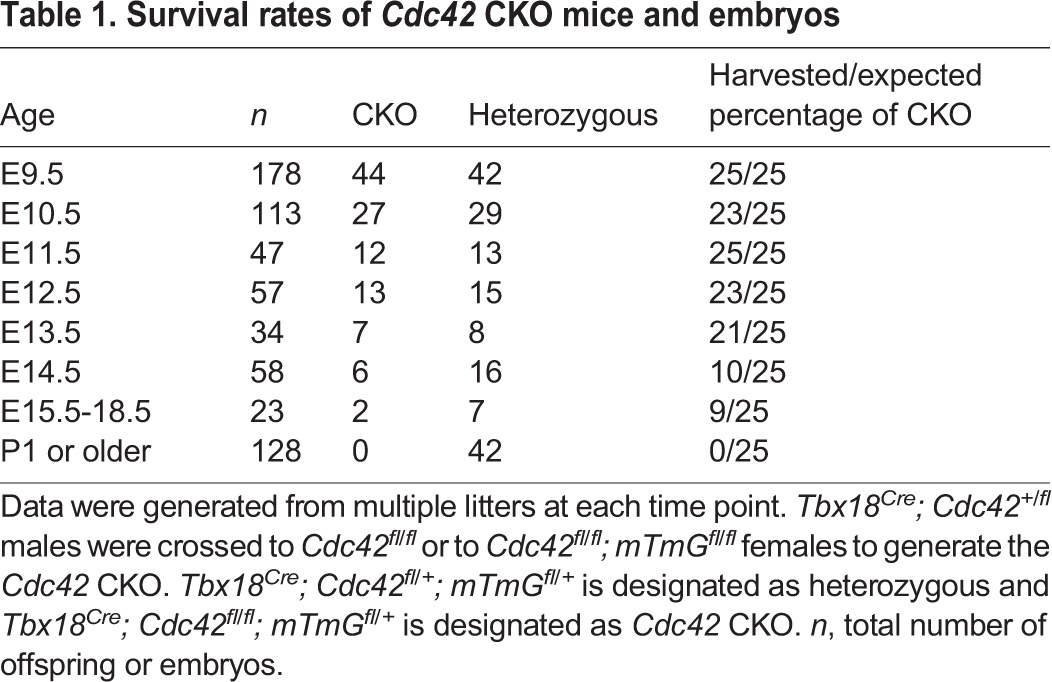
**Survival rates of *Cdc42* CKO mice and embryos**

To thoroughly examine epicardium development and visualize the epicardial covering of the entire heart, ECs were lineage traced via the mTmG reporter system. Hearts of genotype *Tbx18^Cre/+^; mTmG*, in which membrane-localized GFP is expressed upon Cre-mediated recombination, were subject to 3D imaging. In control hearts, both the ventral and dorsal surfaces were fully covered by GFP^+^ cells at E11.5 (Fig. 1G); however, in the CKO heart the ventral surface lacked epicardial coverage, and the dorsal surface was only partially covered (Fig. 1H), consistent with the results observed in sections stained for WT1 (Fig. 1C-E). We examined cell proliferation via BrdU pulse labeling and found that the proliferation rate of ECs in the CKO was higher than that of the control at E12.5, possibly owing to their lower density in the CKO epicardium (Fig. S1B), suggesting that decreased EC proliferation is not the cause of the epicardium developmental defect.

### CDC42 is required for PEC translocation to the myocardial surface

**Fig. 2.**
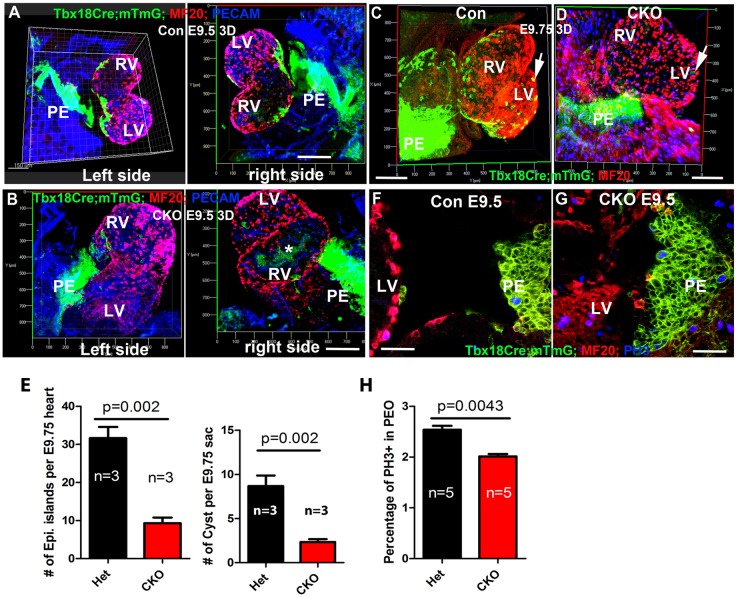
**CDC42 is required for PEC translocation to the myocardial surface.** (A,B) The 3D reconstructed control heart was partially covered with GFP-labeled ECs (A), whereas in the CKO few GFP^+^ cells translocated to the heart surface (B). (C,D) Similarly, control hearts at E9.75 were covered with more ECs and epicardial islands (arrows) than the CKO hearts, based on the reconstructed 3D images. (*Only the GFP^+^ regions of the hearts were imaged. Some signals inside the heart lumen are from the autofluorescence of hematopoietic cells.) (E) The control (Het) contained a significantly greater number of epicardial islands at the surface of the heart and of cysts in pericardial sac than did the CKO. (F-H) The proliferation of PECs was quantified by the percentage of PH3^+^ cells among total GFP^+^ cells in the PE and the heterozygote displayed a significantly higher proliferation rate than the CKO (H). A, atrium. An unpaired two-tailed Student's *t*-test was used to determine statistical significance. Scale bars: 200 μm in A-D; 50 μm in F,G.

We hypothesized that the incomplete epicardial coverage in the CKO is caused by abnormal development of the PE. To study PE development in a spatiotemporal manner, the PEs of control and CKO embryos at different ages were imaged by confocal microscopy and 3D reconstructed with Imaris software. The PE, labeled by GFP, is localized to the surface of the sinus venosus, and is in close proximity to the dorsal wall of the developing ventricles based on the reconstructed images of heart (Fig. 2A,B) and sagittal sections (Fig. S2A,B). GFP^+^ cells partly covered the dorsal surface of the ventricles (Fig. 2A) in the control but not in the CKO at E9.5 (Fig. 2B, Movies 1-4). Epicardial islands were rarely observed on the ventral surface of either control or CKO hearts at this stage (Fig. 2A,B, Movies 1-4).

We then examined the heart and PE at E9.75 and found that the difference between control and CKO was even more dramatic (Fig. 2C,D). The dorsal surface of the control, but not CKO, heart was almost covered by ECs (Fig. 2C,D, Movies 5 and 6), suggesting that translocation of PECs to the heart was disrupted in the CKO. We quantified the numbers of epicardial islands on the myocardial surface and free-floating cysts in the pericardial sac, and found that the CKO had significantly fewer epicardial islands and cysts at E9.75 than the control (Fig. 2E), indicating that CDC42 is required for PEC dissociation. We also examined the expression level of integrin α4 and integrin β1, which are required for PEC attachment to myocardium (Sengbusch et al., 2002; Yang et al., 1995), and found that there was no significant difference between control and CKO (Fig. S2C-F). These results suggest that the reduced number of epicardial islands in the CKO might be caused by a defect in PEC dissociation but not attachment.

**Fig. 3.**
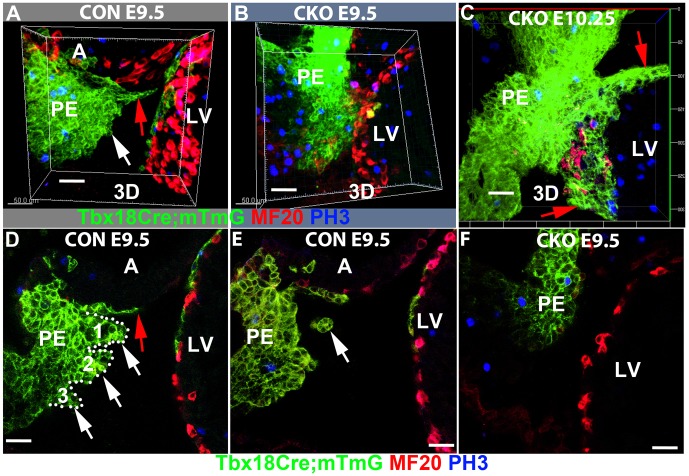
**CDC42 is required for the formation of multicellular villi and cysts.** (A-F) Control (Con) and CKO embryos were whole-mount stained, cleared, imaged and 3D reconstructed using Imaris. The control heart was partially covered with GFP^+^ cells (A), whereas in the CKO fewer GFP^+^ cells translocated to the heart surface (B). Surprisingly, the pro-epicardium (PE) contacted the ventricle directly at this stage in some CKOs (B), and the PE contacted the ventricle at E10.25 in all of the CKOs (C). D and F show one section taken from A and B, respectively. White arrows in D indicate the three villi (1-3), which are demarcated with dotted line; the red arrows indicate the outgrowth of PE along the surface of atrium. (A,E) White arrow indicates a cyst in an E9.5 PE. Scale bars: 40 μm.

We then examined PEC proliferation via whole-embryo staining for phosphorylated histone H3 (PH3), a mitotic marker, and found that the percentage of mitotic PECs was significantly lower in the CKO than in the control (Fig. 2F-H, Fig. 3A-F, Movies 7 and 8). We also examined apoptosis by staining the PE sections with antibody to cleaved caspase 3 and observed no significant difference between control and CKO (data not shown).

### CDC42 is required for the formation of multicellular villous projections and floating cysts

Previous reports demonstrate that PECs translocate to the heart via two mechanisms: budding out from the villi as free-floating cysts (in mammals), or extending bleb-like villi to form a transient tissue bridge that reaches the dorsal surface of the avian heart. We observed no direct contact between PE and heart in six out of ten embryos at E9.5 in the control (Fig. 3A, Fig. S3A), but did observe contact in three out of six CKO embryos (Fig. 3B, Fig. S3B). At E10.25, the PE contacted the heart in three out of four control hearts and in all four of the CKO hearts examined (Fig. 3C), consistent with a previous study (Rodgers et al., 2008). Surprisingly, villi were observed in the mouse heart too, and both villi and floating cysts were formed in the control at E9.5 (*n*=8/8), but significantly fewer cysts (Fig. 2E) and no villi in the CKO heart (*n*=8/8) (Fig. 3D-F). The absence of floating cysts and villi explains why only the dorsal but not the ventral surface of the CKO heart was partially covered by ECs or epicardial islands (Fig. 1C-H), suggesting essential functions of CDC42 in the formation of cysts and villi. We next asked how CDC42 regulates the formation of cysts and villi.

### CDC42 is required to establish PEC polarity

CDC42 regulates actin organization in vascular endothelial cells (Barry et al., 2015) and is required for the formation of spike-like protrusions termed filopodia, which play an essential role in cell migration and cell ruffling (Mattila and Lappalainen, 2008). We observed filopodia in the ECs that attached to the heart, but not in the *Cdc42* null ECs (Fig. S4A,B). However, filopodia were not observed in the control or CKO PECs (data not shown), suggesting that filopodia are not involved in PEC dissociation.

**Fig. 4.**
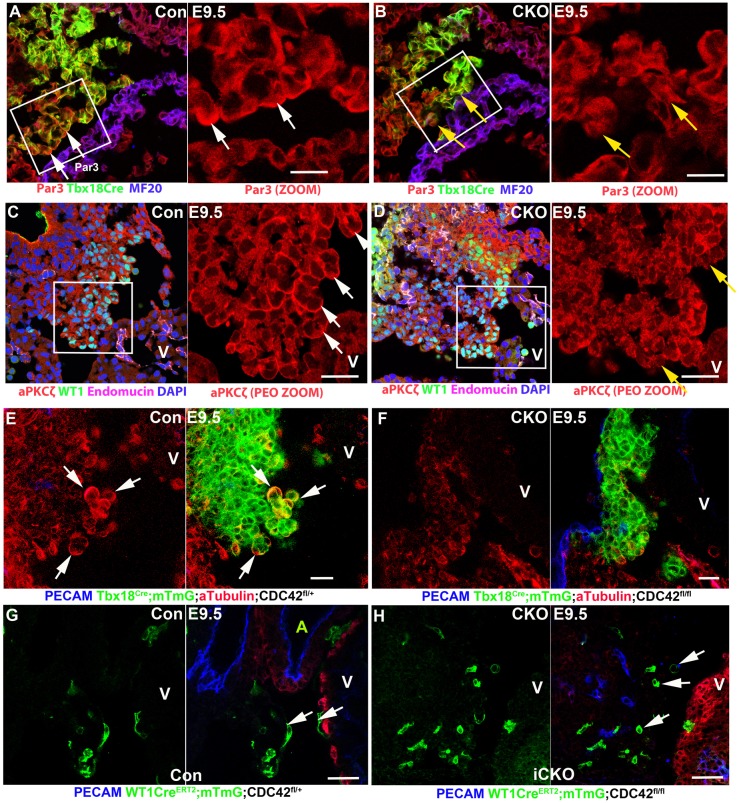
***Cdc42* null cells display loss of polarity and impaired cellular dynamics.** (A,C) Control pro-epicardial cells (PECs) showed Par3 and aPKCζ asymmetric distribution to the apical domain of PECs at the surface of the PE, as indicated by white arrows. (B,D) In the CKO, the asymmetric distribution is not obvious for either protein, as indicated by yellow arrows. (E,F) The control but not the CKO showed enriched acetylated α-tubulin in the PECs or cysts (arrows in E). Dams were gavaged with tamoxifen at a lower concentration at E8.5 for 24 h, and the embryos were harvested at E9.5. (G,H) The labeled control cells displayed an elongated morphology (white arrows, G), whereas most of the *Cdc42* null cells displayed a round shape (white arrows, H). The boxed regions in A-D are magnified on the right. Scale bars: 10 μm in A-D; 20 μm in E-H.

Previous work has shown that Par3 (Pard3) is required for cyst formation by establishing PEC polarity (Hirose et al., 2006). As CDC42 is an essential protein in the establishment of cell polarity (Etienne-Manneville, 2004), we examined the polarity of control and CKO PECs by determining the localization of the polarity complex including Par3 and aPKCζ (PRKCZ), a member of the aPKC family, which is required for epicardial development (Christoffels et al., 2009). Both proteins are more abundant in those PECs that are at the surface of the PE (Fig. 4A-D). Par3 is asymmetrically enriched to the apical domain in the majority of PECs of control hearts (Fig. 4A), but not in the CKO (Fig. 4B). Similarly, aPKCζ is enriched at the membrane cortically in some of the PECs of the control (Fig. 4C), but not of the CKO (Fig. 4D). These results suggest that CDC42 is required to establish PEC polarity, which has previously been shown to be essential for cyst formation in epithelial cells (O'Brien et al., 2002).

It has been reported that CDC42 regulates polarity by activating aPKC, which causes the phosphorylation and inactivation of GSK3β at the leading edges of migrating astrocytes to allow adenomatous polyposis coli to stabilize microtubules at leading edges (Etienne-Manneville and Hall, 2003). We examined stable microtubules, as identified by acetylated α-tubulin, and found that they were abundant in the PECs at the surface of the PE and in floating PECs; 34% of the control PECs and floating cysts (*n*=79 from three hearts; Fig. 4E, Fig. S4C) but only 10% of those from the CKO (*n*=57 from three hearts; Fig. 4F, Fig. S4D) showed asymmetric microtubule distribution.

### *Cdc42* null PECs display impaired dynamics

The PECs undergo active cell movement as they translocate through the pericardial cavity and then spread on the surface of the heart (Komiyama et al., 1987). To study the role of CDC42 in EC morphology and migration, we applied a mosaic model using *Wt1^Cre^^ERT2/+^* and a lower concentration of tamoxifen, which induces sparse recombination, in order to delete *Cdc42* in a few ECs. The control cells showed very diverse and elongated shapes, indicating a dynamic cellular morphology (Fig. 4G), whereas the *Cdc42* null cells were round in shape with a less dynamic cellular morphology (Fig. 4H).

To examine CDC42 functions in cell dynamics and migration in cultured mouse ECs (MEC1; Li et al., 2011), we overexpressed dominant-negative *Cdc42 T17N* (*dnCdc42*) (Jin et al., 2013) in the MEC1 cells to mimic *Cdc42* deletion, and observed that the cells migrated a shorter distance in a wound-healing assay. By contrast, cells that expressed the constitutively active *Cdc42 F28L* (*caCdc42*) (Wu et al., 1998) migrated a greater distance than in the control (Fig. S4E).

### FGF2 promotes epicardial island formation in a CDC42-dependent manner

**Fig. 5.**
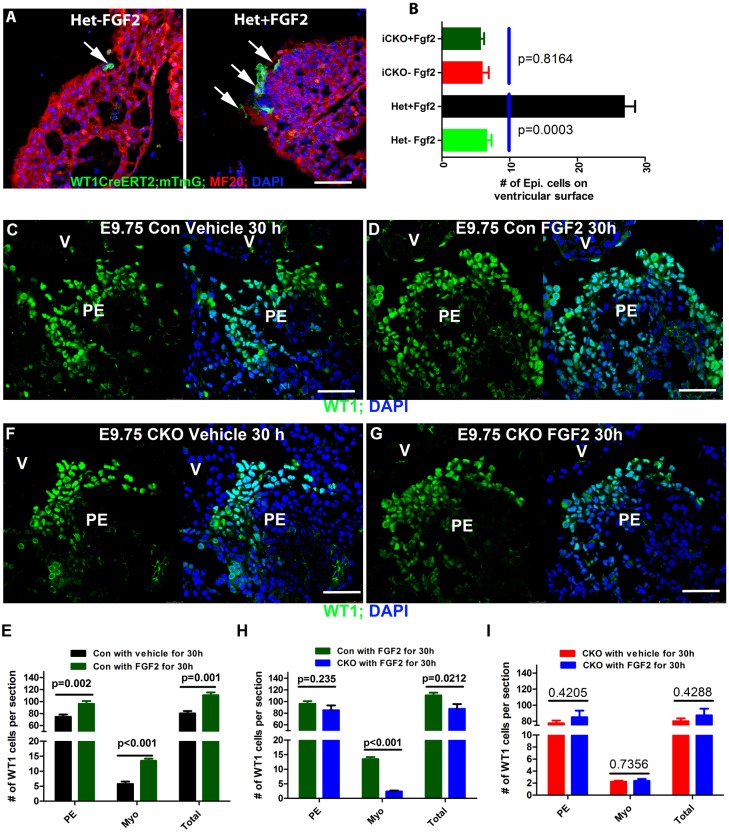
**FGF2 stimulation promotes PEC translocation *ex vivo* and *in vivo*.** (A,B) Heterozygous (Het) and iCKO E9.5 embryos from pregnant females that were gavaged with tamoxifen at E8.5 were cultured with vehicle or FGF2 at 2 ng/ml *ex vivo*. FGF2-stimulated Het hearts showed significantly more GFP^+^ (*Wt1^CreERT2/+^; Cdc42^fl/+^; mTmG*) cells than vehicle-treated hearts. (B) FGF2 stimulation did not rescue the translocation defect of iCKO. Arrows in A point to GFP-labeled epicardial cells. (C-I) Embryos at E9.75 from pregnant females, which were subcutaneously injected with FGF2 at 20 ng/g when the embryos were at E8.5, were sectioned and stained for WT1. Three sections at relatively similar locations in the PE of each embryo, and three embryos for each genotype and each treatment were quantified and are presented in E,H,I. FGF2 stimulation of the control embryos promotes PECs to translocate to the myocardium, and increases the number of WT1^+^ cells in the PE and in total (E); the stimulation did not rescue the translocation defect of the CKO (H,I). One-way ANOVA was used to determine statistical significance. Scale bars: 50 μm.

Previous RNA *in situ* hybridization (ISH) demonstrated that several FGF ligands (FGF2, 10) and receptors (FGFR1, 2, 4) are expressed in the avian PE (Kruithof et al., 2006; Torlopp et al., 2010). Similarly, different FGF receptors display distinct expression patterns in the mouse heart, but their expression in the PE was not determined (Lavine et al., 2005). We determined by ISH that *Fgfr1* is expressed in the murine PE (Fig. S5A,B). To examine whether FGF2 is involved in PEC dissociation in the mouse, we cultured control E9.5 embryos (*Wt1^CreERT2^; Cdc42^fl/+^; mTmG*) from dams that were induced with tamoxifen at E8.5 with either vehicle or FGF2 at 2 ng/ml for 24 h. FGF2-treated embryos showed significantly more ECs on the heart surface than vehicle treated embryos (Fig. 5A,B, Movies 9 and 10). We then examined whether *ex vivo* FGF2 treatment would rescue the PEC dissociation and translocation defects in *Wt1^CreERT2^; Cdc42^fl/fl^; mTmG* (iCKO) embryos. FGF2 treatment for 24 h did not increase the number of ECs on the heart surface as compared with vehicle-treated iCKO hearts (Fig. 5B).

To determine whether FGF2-mediated signaling promotes PEC translocation to the heart surface *in vivo*, we treated the dams when the embryos were at E8.5 with FGF2 at 20 ng/g body weight by subcutaneous injection and examined pro-epicardial development 30 h later at E9.75. We quantified the numbers of WT1^+^ cells located in the PE, attached to the myocardium, and in total for embryos of the different genotypes and treatments. These data showed that the number of WT1^+^ cells in FGF2-treated embryos was significantly greater than for the vehicle control (Fig. 5C-E). Furthermore, we found that the FGF2-stimulated CKO displayed a significant reduction in the number of WT1^+^ cells that attached to the myocardium or in total (Fig. 5F-H), and a trend to a smaller number of WT1^+^ cells in the PE than the FGF2-treated control (Fig. 5D,G,H). To determine whether FGF2 stimulation would promote PEC dissociation and translocation in the CKO, the numbers of WT1^+^ cells in the PE and on the myocardium were compared between CKOs treated with vehicle or FGF2, and were found not to be significantly different (Fig. 5F,G,I). Furthermore, that FGF2 stimulation could promote PEC translocation to myocardium in the control heart, but could not rescue the dissociation defect in CKO, was confirmed using the mTmG reporter system (Fig. S5C,D). These results suggest that CDC42 is involved in FGF2-mediated PE expansion, and in PEC dissociation and translocation *in vivo*.

### CDC42 is required for the intracellular trafficking of FGF receptors

**Fig. 6.**
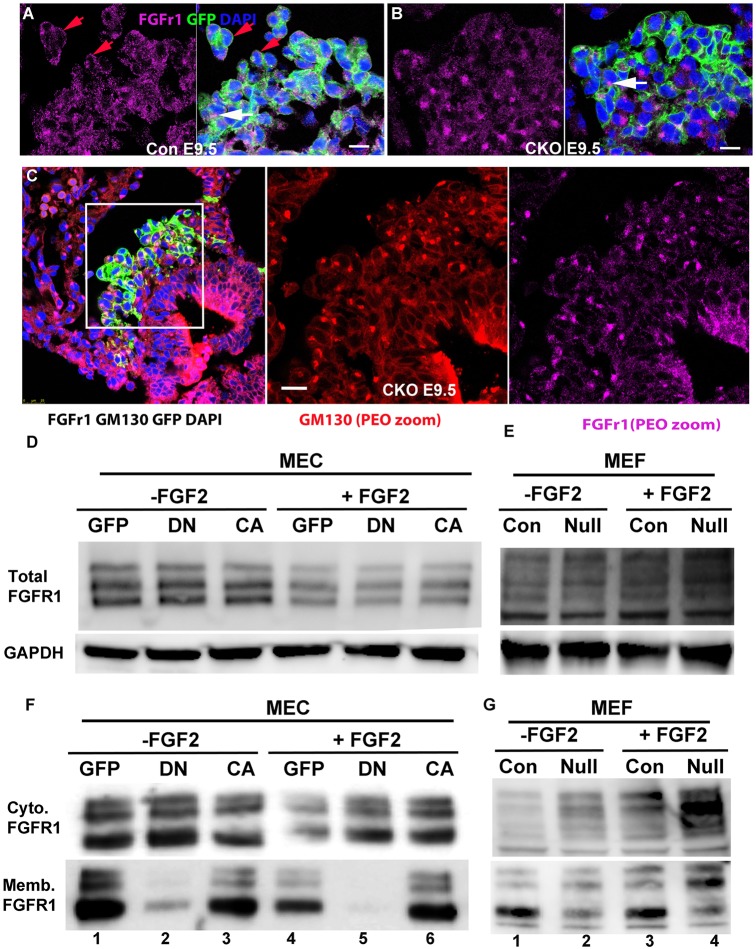
**CDC42 is required for FGFR1 trafficking.** (A,B) FGFR1 localizes to the membrane in most of the cells in the control (A), whereas FGFR1 is enriched to the perinuclear region in most PECs of the CKO (B). White arrows in A,B indicate the perinuclear localization of FGFR1 and red arrows indicate the membrane localization of FGFR1. (C) FGFR1 and GM130, a marker for Golgi, were colocalized in the CKO (boxed area is enlarged in the middle and left panels). (D) MEC1 was cultured and then infected with GFP, or *dnCdc42* (DN) or *caCdc42* (CA) virus for 36 h, starved overnight and then stimulated with FGF2 at 2 ng/ml for 15 min. (E) Similarly, *Cdc42* control and null MEFs were starved and treated as the MEC1. Total lysates were harvested and were used for western blot. (D,E) The total levels of FGFR1 in GFP-, DN- and CA-infected cells or in control and *Cdc42* null MEFs, whether they were treated with FGF2 or vehicle, are not significantly different. Cells with similar treatments to the experiment in D,E were starved and dissociated to single cells, and then stimulated with FGF2 for 15 min. (F,G) The cells were collected and used to separate the cytosol and membrane fractions via biotinylation. We found that FGFR1 localized to membrane and cytosol in controls (lane 1 in F,G), but mainly localized to cytosol in *dnCdc42*-infected MEC1 cells or *Cdc42* null MEFs (lane 2 in F,G). FGF2 stimulation did not promote FGFR1 translocation to membrane (lanes 4 and 5 in F, lanes 3 and 4 in G). The membrane and cytosolic fractions were normalized to GAPDH, and the ratios of membrane to cytosolic fraction were quantified. This experiment was repeated three times and the quantified results are stated in the Results. Scale bars: 10 μm in A,B; 20 μm in C.

To study how CDC42 mediates FGF2 signaling during PEC dissociation and translocation, the expression patterns of four potential receptors (FGFR1-4) in PECs were examined. FGFR1 localized to the plasma membrane in most cells, although it accumulated in the perinuclear region in some cells (Fig. 6A). Surprisingly, FGFR1 localized to the perinuclear region in most PECs of the CKO (*n*=3; Fig. 6B). The expression of *Fgfr1* mRNA in the PE was confirmed by ISH, and there was no obvious difference between control and CKO (Fig. S5A,B). We then co-stained the PE for FGFR1 and GM130 (GOLGA2), a marker for the Golgi, and found that FGFR1 colocalized with GM130 (Fig. 6C).

To further study FGFR1 subcellular localization, we examined MEC1 cells that overexpressed *dnCdc42*, as well as *Cdc42* null mouse embryonic fibroblasts (MEFs). We found that a large portion of FGFR1 colocalized with GM130 in *dnCdc42*-expressing MEC1 cells or *Cdc42* null MEFs (Fig. S6A). Western blotting was then used to determine the relative levels of FGFR1-4 between *caCdc42-* and *dnCdc42*-overexpressing MEC1 cells or between control and *Cdc42* null MEFs (Fig. 6D,E). The data revealed that *dnCdc42-*expressing MEC1 cells or *Cdc42* null MEFs produced similar levels of FGFR1 to the control cells (Fig. 6D,E). We also found that each of the samples contained similar isoforms of FGFR1 to its control, whether treated with vehicle or FGF2 (Fig. 6D), indicating that FGFR1 expression, maturation and isoform splicing are not affected by FGF2 stimulation or *Cdc42* deletion.

We hypothesized that CDC42 might be involved in FGFR1 trafficking from the Golgi to the membrane during FGF ligand stimulation. We fractionated the membrane and cytosolic FGFR1 from adherent cells that were treated with FGF2 or vehicle. At baseline, little FGFR1 localized to the membrane in MEC1 cells; however, with FGF2 stimulation, FGFR1 translocated to membrane in the control but to a substantially lesser extent in *dnCdc42*-expressing MEC1 cells (Fig. S6B), indicating that CDC42 is involved in FGFR1 trafficking to the cell membrane. When MEC1 cells and MEFs were dissociated to individual cells, a significant portion of FGFR1 localized to membrane, and the control showed significantly more FGFR1 localized to membrane than the *dnCdc42*-expressing MEC1 cells (Fig. 6F,G). The ratio of FGFR1 in the membrane versus cytoplasmic fraction was 1.2±0.3 (*n*=3) in the control and 0.2±0.1 (*n*=3) in *dnCdc42*-infected cells. Consistently, the ratio of FGFR1 in the membrane versus cytoplasmic fraction was 1.5±0.2 (*n*=3) in the control and 0.9±0.2 (*n*=3) in *Cdc42* null MEFs (Fig. 6F,G). Surprisingly, when cells were dissociated, FGF2 stimulation did not increase the membrane localization of FGFR1 in *dnCdc42* MEC1 cells or *Cdc42* null MEFs (Fig. 6F,G). These results suggest that whether cells were attached to a plate or dissociated to individual cells, CDC42 regulates FGFR1 trafficking to membrane.

The localization patterns of FGFR2-4 between control and CKO were not notably different, but showed slight differences with weaker membrane localization for FGFR2 and FGFR3 in the control compared with CKO (Fig. S6C-E). The protein levels of FGFR2-4 in *Cdc42* null MEFs were significantly lower than in the control (Fig. S6F). The transcriptional levels of *Fgfr1-4* were not significantly different between control and *Cdc42* null MEFs (data not shown), indicating that the lower protein levels of the receptors were not a result of transcriptional regulation but were likely to be due to impaired trafficking in *Cdc42* null cells (Harris and Tepass, 2010).

### CDC42 is required for FGF2-mediated signaling in PE and PEC proliferation

**Fig. 7.**
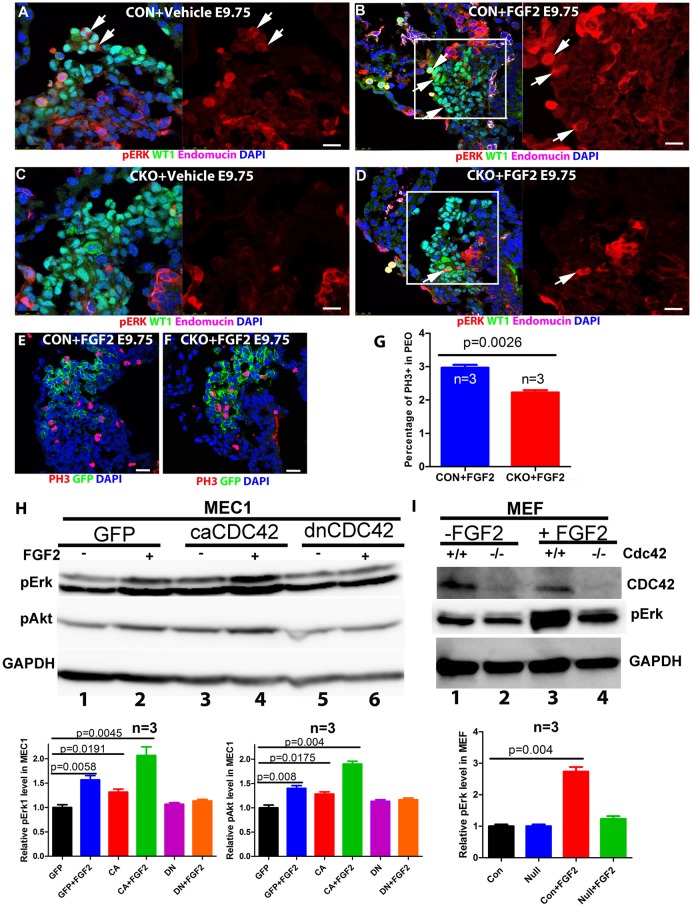
**FGF2 promotes Erk and Akt phosphorylation, and PEC proliferation in a CDC42-dependent manner.** (A-D) The FGF2-stimulated controls showed more pErk^+^ cells than the vehicle-treated controls (A,B), and FGF2 stimulation did not increase the number of pErk^+^ cells in the CKO (C,D). Boxed areas are magnified on the right. (E-G) FGF2 treatment in the control *in vivo* significantly increases the percentage of PH3^+^ cells in the PE compared with the same treatment in the CKO. (H,I) MEC1 cells were cultured and infected with GFP, *dnCdc42* or *caCdc42* virus for 36 h, starved overnight and then stimulated with vehicle or FGF2 at 2 ng/ml for 15 min. FGF2 stimulation increased pErk and pAkt in the GFP-infected but not *dnCdc42*-infected cells (lanes 2 and 6 in H). The baseline pErk level is increased in the *caCdc42*-infected cells, and increased even more with FGF2 stimulation (lanes 1, 3 and 4 in H). Control and *Cdc42* null MEFs were treated in a similar way, and the FGF2 stimulation in *Cdc42* null MEFs increased the pErk protein level but not significantly (lanes 2 and 4 in I), whereas FGF2 stimulation in control MEFs dramatically increased pErk protein level (lanes 1 and 3 in I). The experiments in H,I were repeated three times and quantified. One-way ANOVA was used to determine statistical significance. Scale bars: 20 μm in A-D; 50 μm in E,F.

To study whether CDC42 is required for PECs to respond to FGF2 stimulation *in vivo*, PECs from control and CKO embryos treated with either vehicle or FGF2 were stained for phosphorylated (p) Erk (Mapk3/1), which functions as a readout for FGF2 signaling (Pintucci et al., 2002). FGF2-treated control PE contained significantly more pErk^+^ cells (21±3%, *n*=3 hearts and total 621 cells) than the vehicle-treated control (12±2%, *n*=3 hearts and total 445 cells, *P*=0.0124), suggesting that FGF2 stimulation promotes Erk phosphorylation in the PE (Fig. 7A,B). The PECs of the FGF2-stimulated CKO (6±2%, *n*=3 hearts and total 557 cells) did not contain a significantly different number of pErk^+^ cells to the vehicle-treated CKO (5±2%, *n*=3 hearts and total 459 cells, *P*=0.5734) (Fig. 7C,D), suggesting that CDC42 is required for Erk phosphorylation following FGF2 stimulation. The *ex vivo* cultured hearts were also examined, and the control PECs displayed a significantly greater percentage of pErk^+^ cells (15±3%, *n*=3 and total 356 cells) than the iCKO (3±1%, *n*=3 and total 378 cells, *P*=0.0028). As BMP signaling is involved in PE translocation to myocardium in chick (Ishii et al., 2010), we stained for pSMAD1,5,8, a readout for BMP signaling, and found no significant differences between control and CKO (Fig. S7A-C) or between control and *Cdc42* null MEFs (Fig. S7D). Taken together, these results support the hypothesis that CDC42 is involved in FGF2-mediated phosphorylation of Erk.

FGF ligands have been suggested to act as autocrine or paracrine growth factors to prevent apoptosis, maintain proliferation, and promote villous outgrowth of the PEC in the avian heart (Torlopp et al., 2010). We examined PEC polarity once again, and found that FGF2 treatment did not rescue the polarity defect of PECs in the CKO, and we did not observe differences in apoptosis between vehicle- and FGF2-treated PECs (data not shown), indicating a species-dependent function of FGF2 in PE. Instead, the FGF2-treated PECs displayed a higher proliferation rate than cells in the vehicle-treated embryos, based on the percentage of mitotic cells (Fig. 7E-G), consistent with the result that PECs in the CKO had a lower proliferation rate than the control (Fig. 2F-H).

To investigate further the role of CDC42 in FGF2-mediated signaling, MEC1 cells were infected with control GFP, *caCdc42* or *dnCdc42* virus and then treated with vehicle or FGF2 for 15 min. *caCdc42*-infected cells, along with the GFP virus-infected cells that were stimulated with 2 ng/ml FGF2, displayed higher levels of pErk and pAkt than their controls, indicating that CDC42 activation or FGF2 stimulation promotes Erk and Akt phosphorylation (Fig. 7H). In *dnCdc42*-infected cells, Erk and Akt phosphorylation was not elevated in response to FGF2 treatment as compared with the vehicle (Fig. 7H). Similarly, *Cdc42* deletion abolished the FGF2-mediated Erk phosphorylation in MEFs (Fig. 7I). These results suggest that CDC42 is required for FGF2-mediated signaling activation.

## DISCUSSION

### Cellular mechanism of PECs translocation to the myocardium

**Fig. 8.**
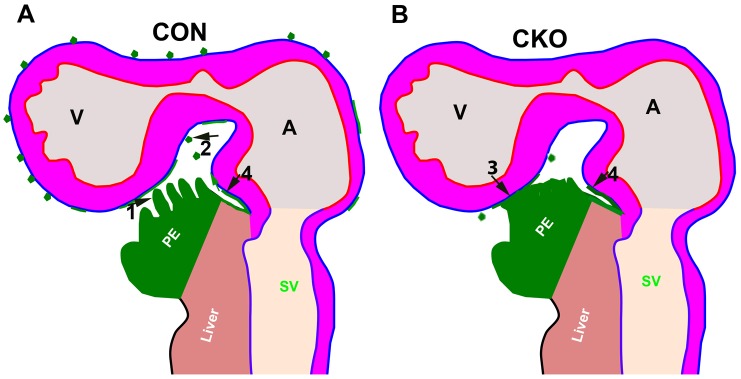
**Mechanisms of PEC translocation to heart.** (A) In the control heart, the PECs employ villous protrusion (1), cyst formation (2), direct contact with myocardium (3, not shown) and migration along the surface of inflow tract (4) to reach the heart. (B) In the CKO, villous protrusion and cyst formation was impaired, and the PECs migrated along the surface of inflow tract (4) and were in direct contact with myocardium (3) to reach the ventricle.

The cellular mechanisms of PEC translocation from the PE to the myocardium are species dependent. In avian embryos, the PE extends bleb-like villi that form a transient tissue bridge to reach the heart, and in mouse the PECs are thought to reach the myocardium through the translocation of multicellular cysts across the pericardial cavity (Hirose et al., 2006; Komiyama et al., 1987; Pérez-Pomares et al., 1997; Ratajska et al., 2008; Schulte et al., 2007; Sengbusch et al., 2002; Van den Eijnde et al., 1995). Another study in the mouse demonstrated that PEC translocation to the heart is achieved by the PE directly contacting the heart (Rodgers et al., 2008). We used lineage tracing, whole-mount staining and 3D imaging to reveal these mechanisms and resolve the controversy. PE cells labeled with GFP via *Tbx18^Cre/+^; mTmG* were observed as early as E8.75 (somite stage 14). At this stage, we did not observe any floating cysts in the pericardial lumen or on the heart surface, and the PE did not physically contact the myocardium in either control or CKO (data not shown). At E9.5, we observed the formation of both free-floating cysts and villous projections (Fig. 3D,E, Fig. 8A,B), which has not previously been reported in the mouse. Interestingly, the formation of floating cysts and villous projections was disrupted in the *Cdc42* CKO (Fig. 3F, Fig. 8A,B). This indicates that the mouse PE forms villi and cysts in a CDC42-dependent manner. Furthermore, the PE directly contacted the heart in four out of ten control embryos (Fig. 3A, Fig. S2A-C, Fig. S3A,B) and in three out of six CKO embryos (Fig. 3A,B, Movie 11) examined at E9.5, and in three out of four control hearts at E9.75, validating a previous report of PE direct contact with the myocardium (Movie 12) (Rodgers et al., 2008), which is referred to as the third mechanism in this study. These results indicate that PECs do not translocate to the heart via direct contact at an early stage, but do so at a later stage; therefore, the controversy might be due to the different ages of the hearts examined. Surprisingly, we discovered a fourth mechanism for PEC translocation to myocardium that has not previously been reported in the mouse but was observed in chick under surgery-induced pathological conditions (Manner, 1999). In this fourth mechanism, the PECs grow from the sinus venosus towards the heart along the surface of the inflow tract (Fig. 3A, Fig. 8A,B, Fig. S2, Fig. S3C). In the CKO, the PE fails to form cysts or villi, but the PECs can migrate along the heart surface (Fig. S2, Fig. S3C, Fig. 8A,B) and the PE can physically associate with the dorsal myocardium (Fig. S3A,B). The two mechanisms allowed PECs to spread and cover the atria and dorsal ventricle, but were insufficient to cover the ventral surface of the heart in the CKO. This incomplete epicardial covering eventually resulted in embryonic lethality, suggesting that the formation of cysts is required for the epicardium to cover the ventral surface of the heart.

Although the routes of translocation uncovered with the Cre lines used in our studies might also be used by other subpopulations, such as those labeled by Scl-Cre or Sema3D-Cre, further investigations will be necessary to establish the generality of these routes for all PECs.

### CDC42 might be required for microtubule-mediated PEC directional migration

In all four cellular mechanisms by which PECs translocate to the myocardium and spread over the surface of the heart, the PECs undergo active cell movement evidenced by the formation of ruffles and pseudopods on ECs (Ho and Shimada, 1978) (Fig. S4A,B). The reduced number of epicardial islands on the ventral surface of the CKO heart can be attributed to disrupted directional guidance or directional migration of PECs. We found that components of the polarity complex, including aPKC and Par3, and stable microtubules accumulated toward the apical domain of the PECs in the control but not CKO. It has been reported that CDC42 regulates polarity by activating aPKC via Par6 (Pard6), leading to phosphorylation and inactivation of GSK3β at the leading edges of migrating astrocytes, which allows APC to stabilize microtubules at leading edges (Etienne-Manneville and Hall, 2003). Furthermore, inhibition of CDC42 prevents the reorientation of the T-cell microtubule-organizing center towards antigen-presenting cells (Stowers et al., 1995), the directional movement of macrophages towards a chemotactic signal (Allen et al., 1998) and the directional movement and reorientation of the fibroblast Golgi apparatus in a wound-healing assay (Nobes and Hall, 1999). Microtubule network polarity establishes and maintains the spatial and temporal coordination of migration events, which is the key to persistent directed migration (Etienne-Manneville, 2013).

In the PE, CDC42 might be required for the stabilized microtubules to localize asymmetrically and for the directional and persistent migration of PECs. This explains why the CKO lacks epicardial islands on the ventral surface of the myocardium, which is further from the PE and requires more persistent migration over a longer distance than for PECs to reach the dorsal surface.

### CDC42 might regulate cellular component trafficking during PE development

The recent discovery of CDC42 in the regulation of endocytosis and recycling (Harris and Tepass, 2010; Osmani et al., 2010) raises the possibility that CDC42 might regulate different signaling pathways via the endocytosis and recycling of their receptors. FGF signaling is essential for mesoderm patterning through the development of multiple organ systems (De Moerlooze et al., 2000; Kimelman and Kirschner, 1987). There are 18 FGF ligands and four receptors in mammals (Turner and Grose, 2010), which might explain the additive effects of FGFR1 and FGFR2 in epicardium development (Pennisi and Mikawa, 2009; Rudat et al., 2013; Vega-Hernandez et al., 2011). In the chick heart, multiple FGF ligands and receptors have been found in the mesenchymal layer and epithelial layer, and FGF signaling promotes villous outgrowth, prevents apoptosis and maintains proliferation (Torlopp et al., 2010). In the mouse, little is known about the FGF signaling pathways that regulate PEC dissociation and translocation. Through ISH and immunostaining, we found multiple FGF receptors in the PECs. FGF2 stimulation promoted Erk phosphorylation *ex vivo* and *in vivo*, and also promoted PEC proliferation, translocation and association with the myocardium, indicating that FGF signaling is involved in PE and epicardium development. In the CKO, FGF2 stimulation failed to promote PEC translocation, association with myocardium and Erk phosphorylation, indicating that CDC42 is required for FGF2-mediated stimulation. Without FGF2 stimulation, the PE in the CKO had fewer pErk^+^ cells, indicating that endogenous FGF ligands regulate PEC proliferation in a CDC42-dependent manner too. The fact that the CKO has fewer pErk^+^ cells *in vivo* and that FGF2 stimulation did not increase the percentage of pErk^+^ cells in the CKO but did so in the control suggest that CDC42 functions downstream of FGF *in vivo*. Our results showed that in the CKO, FGFR1 membrane localization was disrupted with corresponding accumulation in the Golgi complex, and that FGFR2 and FGFR3 showed less membrane localization, indicating that CDC42 is required for FGF receptors to traffic to the plasma membrane. The disrupted trafficking of FGFR2-4 might contribute to the reduction in protein levels (Fig. S6F). This provides *in vivo* evidence that CDC42 is involved in the trafficking of receptors from the Golgi to the membrane, and is consistent with a report that the Golgi complex represents a predominant location for CDC42 in mammalian cells (Farhan and Hsu, 2016). The current study is consistent with the finding that CDC42 regulates bidirectional Golgi transport by targeting the dual functions of Coat protein complex I in cargo sorting and carrier formation (Park et al., 2015).

That FGF2 stimulation did not rescue cell polarity and PEC dissociation defects indicates that CDC42 functions encompass more than FGF receptor recycling, and CDC42 might function as a hub to regulate multiple steps in PEC dissociation and translocation: CDC42 is required for recycling polarity proteins to the cell cortex to establish PEC polarity, for FGF receptor trafficking to the membrane, and for the asymmetric localization of stable microtubules in order to direct persistent cell migration.

In summary, this study demonstrates that CDC42 plays a role in epicardial and pro-epicardial development by controlling the formation of villi and free cysts, possibly through regulation of cell polarity and microtubule organization, and that CDC42 regulates cell proliferation through mediating FGF signaling, where CDC42 is required for FGFR1 trafficking from the Golgi to the cell membrane in PECs. This study reveals that CDC42 adds another layer to the complex regulation of FGF signaling by controlling FGFR1 trafficking. Furthermore, this study indicates that FGF2 might be a therapeutic candidate with a view to enhancing epicardial proliferation and epicardial-mediated regeneration during injury.

## MATERIALS AND METHODS

### Mouse strains and cell lines

Mouse strains *Gt(ROSA)26Sor^tm4(ACTB-td^^Tomato,-^^EGFP^^)^* (*mTmG*) (Muzumdar et al., 2007) and *Wt1^CreERT2^* (Zhou et al., 2008) were purchased from The Jackson Laboratory. *Cdc42^fl/fl^* was a gift from Dr Yi Zheng (Chen et al., 2006). *Tbx18^Cre/+^* was a gift from Dr Chenleng Cai (Cai et al., 2008). *Tbx18^Cre^; Cdc42^+/fl^* males were crossed to *Cdc42^fl/fl^* or *Cdc42^fl/fl^; mTmG^fl/fl^* females to generate the CKO. *Tbx18^Cre/+^; Cdc42^fl/+^; mTmG^fl/+^* and *Tbx18^Cre^;* *Cdc42^fl/fl^;* *mTmG^fl/+^* were designated as heterozygous or control and as CKO, respectively. The inducible *Wt1^CreERT2/+^* line was used to delete *Cdc42* to generate *Wt1^CreERT2/+^; Cdc42^fl/+^; mTmG*, which is designated as iCKO. Embryos harvested at around noon on embryonic day 9 were counted as E9.5, and those harvested at ∼6 pm were counted as E9.75. *Ex vivo* culture is decribed in the supplementary Materials and Methods. All animal experiments are approved by the Institutional Animal Care and Use Committee at Albany Medical College and performed according to the NIH Guide for the Care and Use of Laboratory Animals.

### Immunofluorescence, *in situ* hybridization and western blot

Immunofluorescence, ISH, western blot and whole-mount staining were performed as previously described (Li et al., 2016; Shaikh Qureshi et al., 2016). Additional details, including imaging and antibodies, are provided in the supplementary Materials and Methods and Table S1.

### Membrane and cytoplasmic fractionation

Membrane and cytoplasmic fractionation was performed according to the protocol provided with the Sulfo-NHS-SS-Biotin Kit (Thermo Fisher Scientific). Briefly, primary MEFs or MEC1 cells (Li et al., 2011) were cultured to confluence and then starved overnight. The cells attached to plate or suspended in PBS were stimulated with FGF2 at 2 ng/ml or vehicle for 15 min, and then treated with 80 μl 10 mM EZ-Link Sulfo-NHS-SS-Biotin to label membrane proteins. NeutrAvidin agarose resins were used to pull down the membrane fraction; the remaining solution constituted the cytoplasmic fraction. These fractions were used to determine relative protein levels in membrane and cytoplasm by western blot. Details of the fractionation procedure and western analysis are provided in the supplementary Materials and Methods and Table S1.

Lineage tracing and mosaic analyses were performed as previously described (Zhou and Pu, 2012) except that Cre induction was performed at a different age and with a lower concentration of tamoxifen. EC proliferation was measured by BrdU pulse labeling as described (Zhao et al., 2014). Additional details of lineage tracing and BrdU labeling are provided in the supplementary Materials and Methods.
